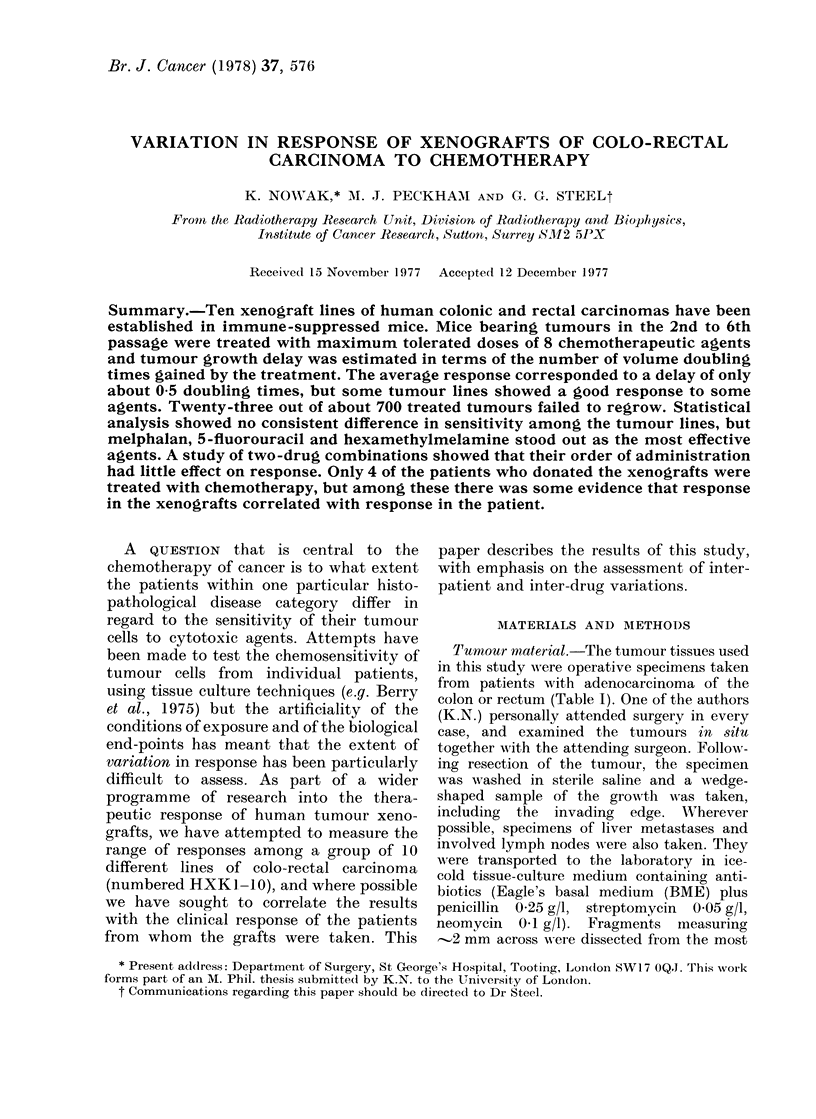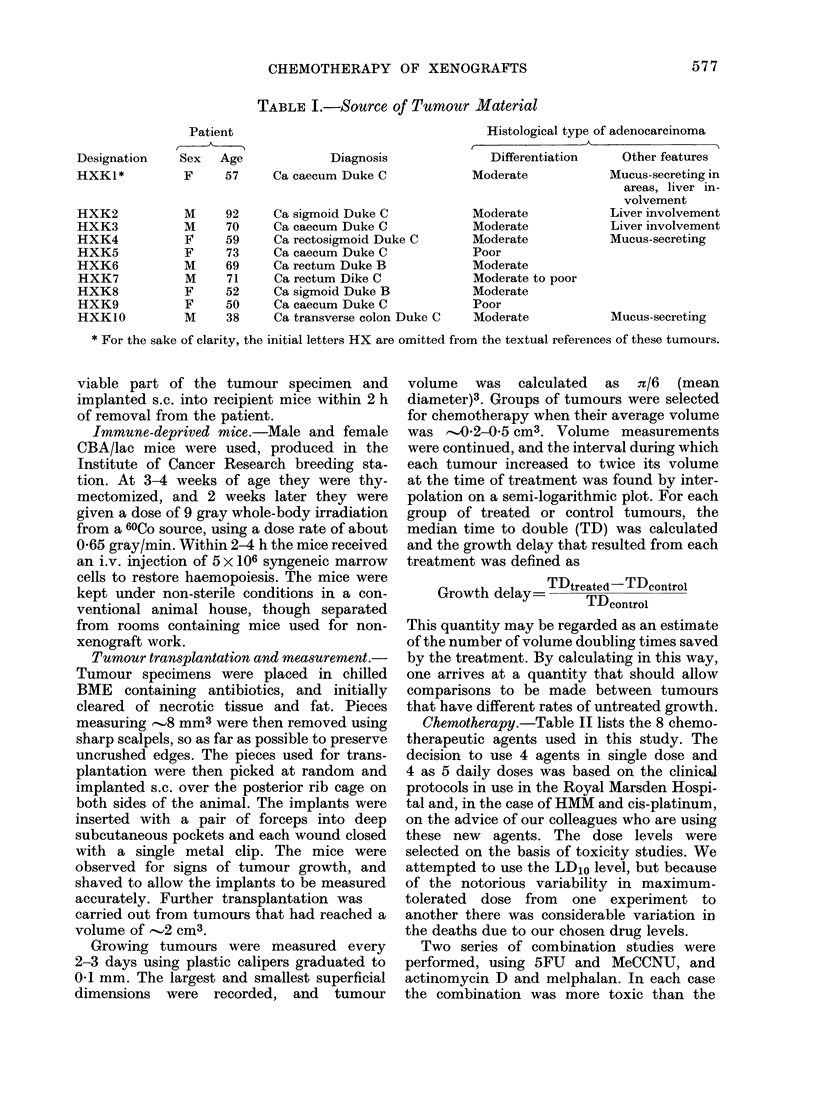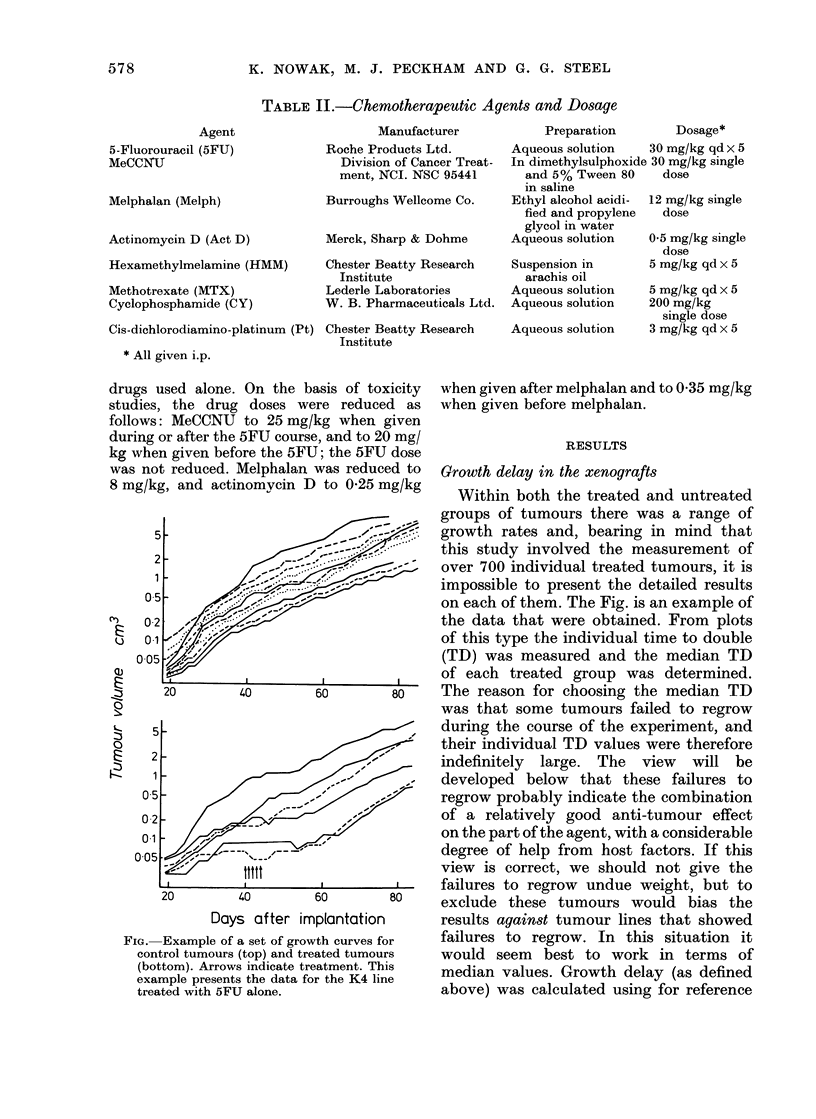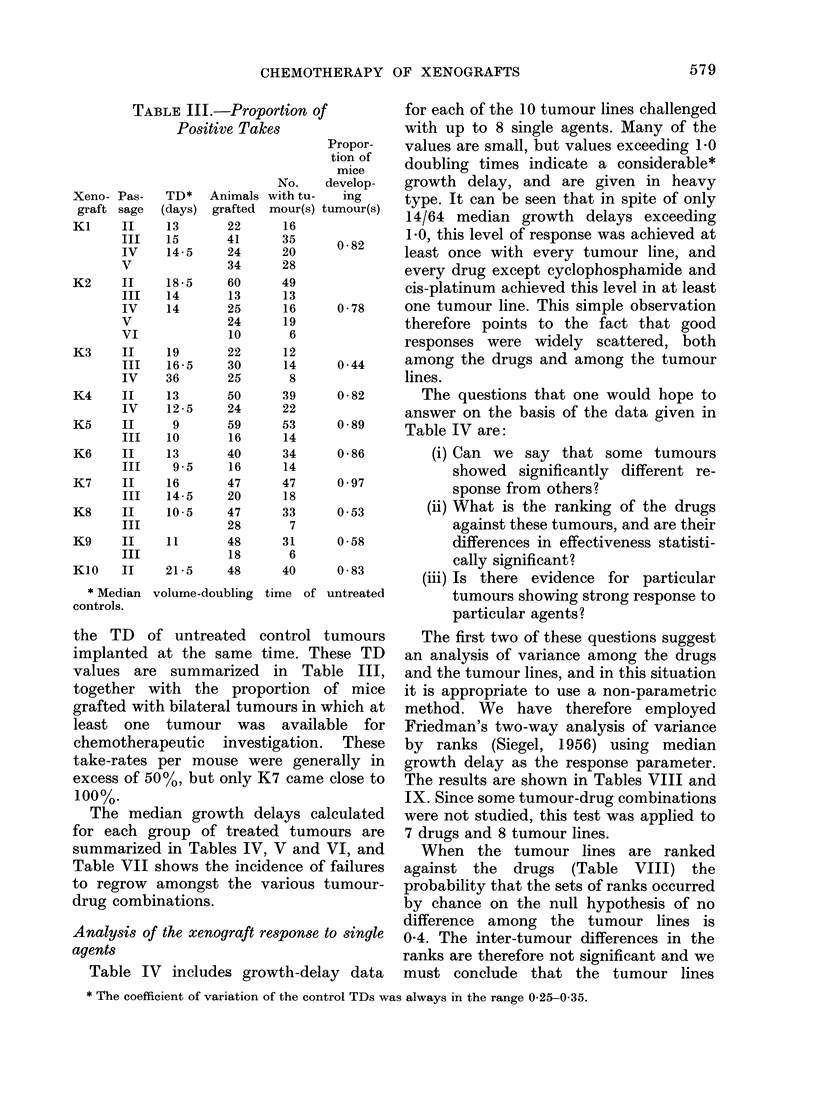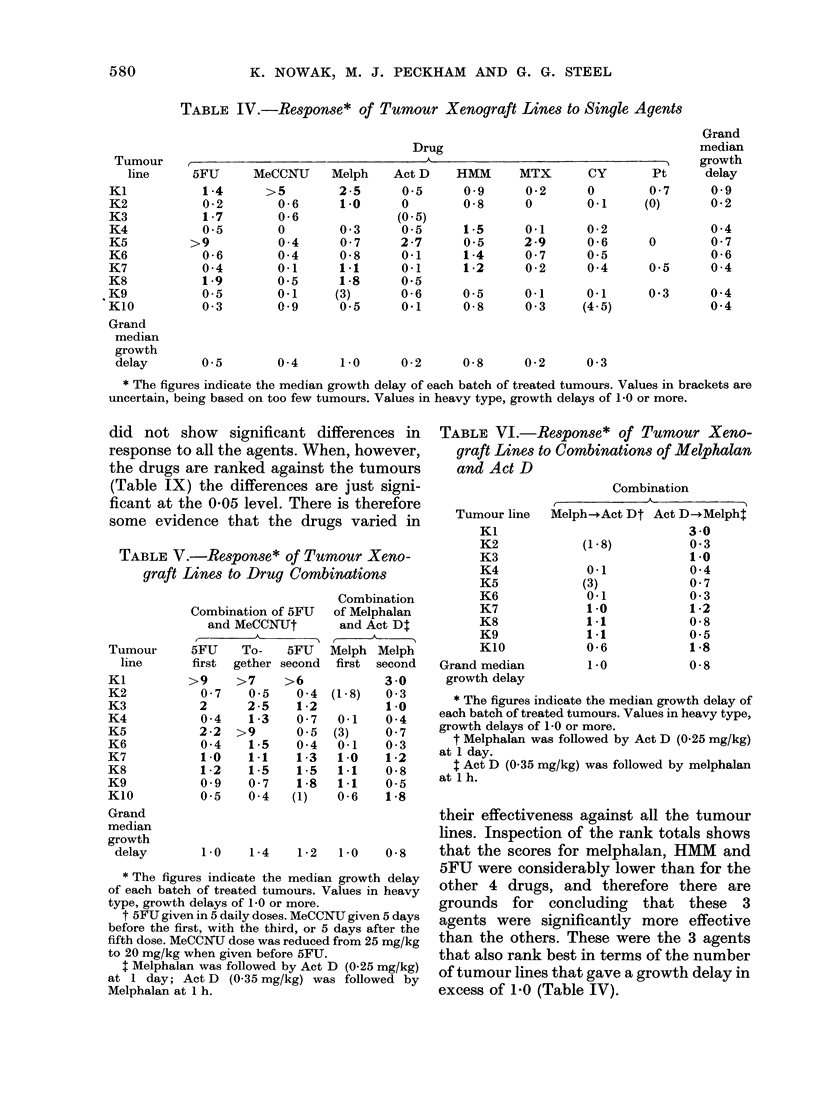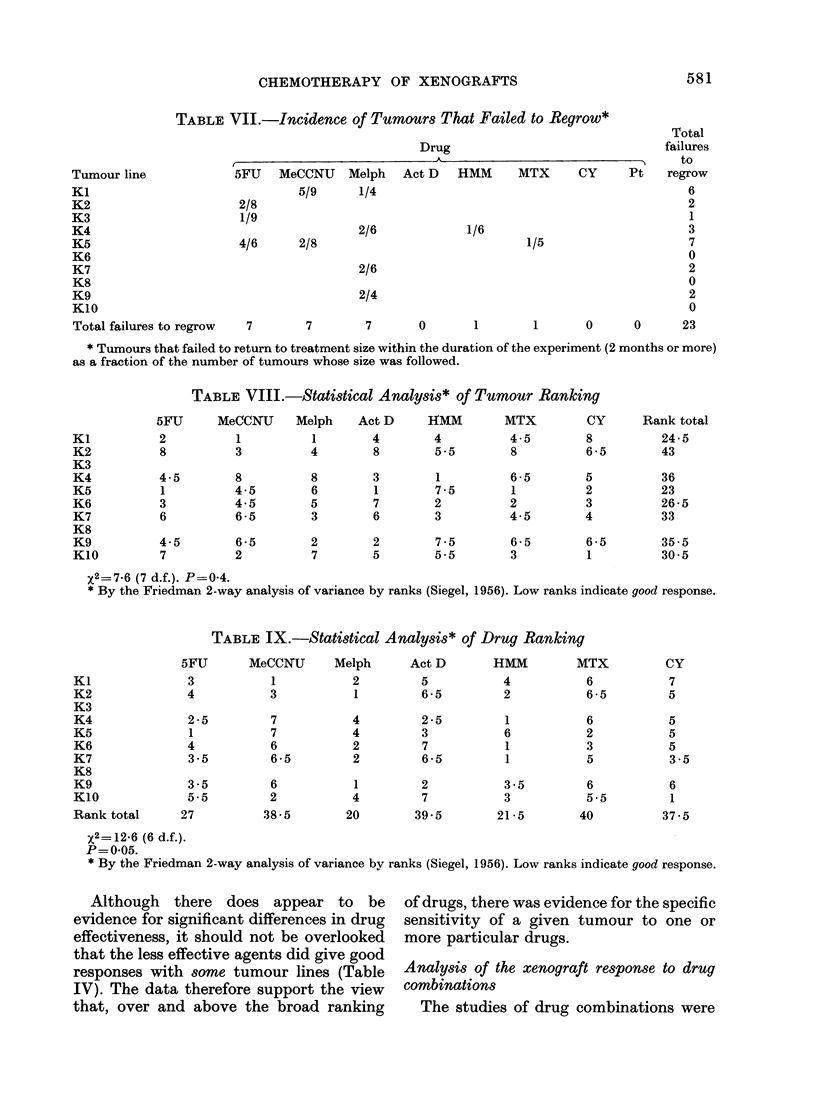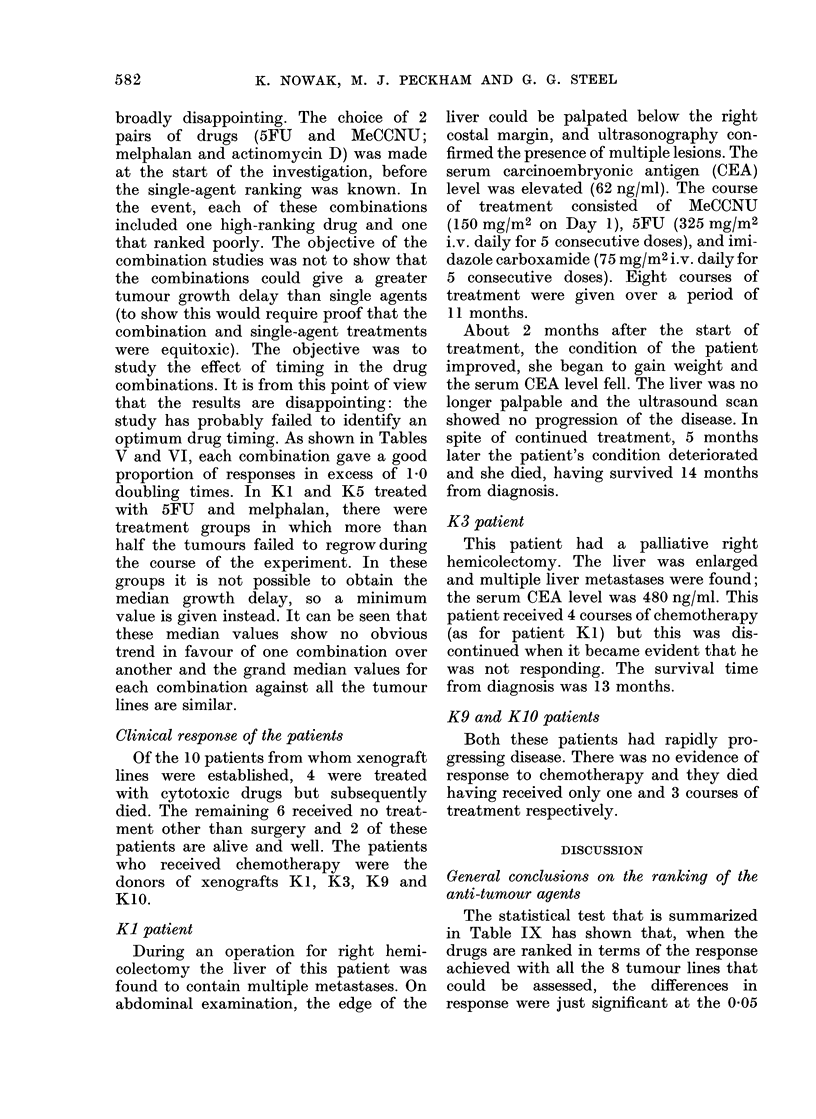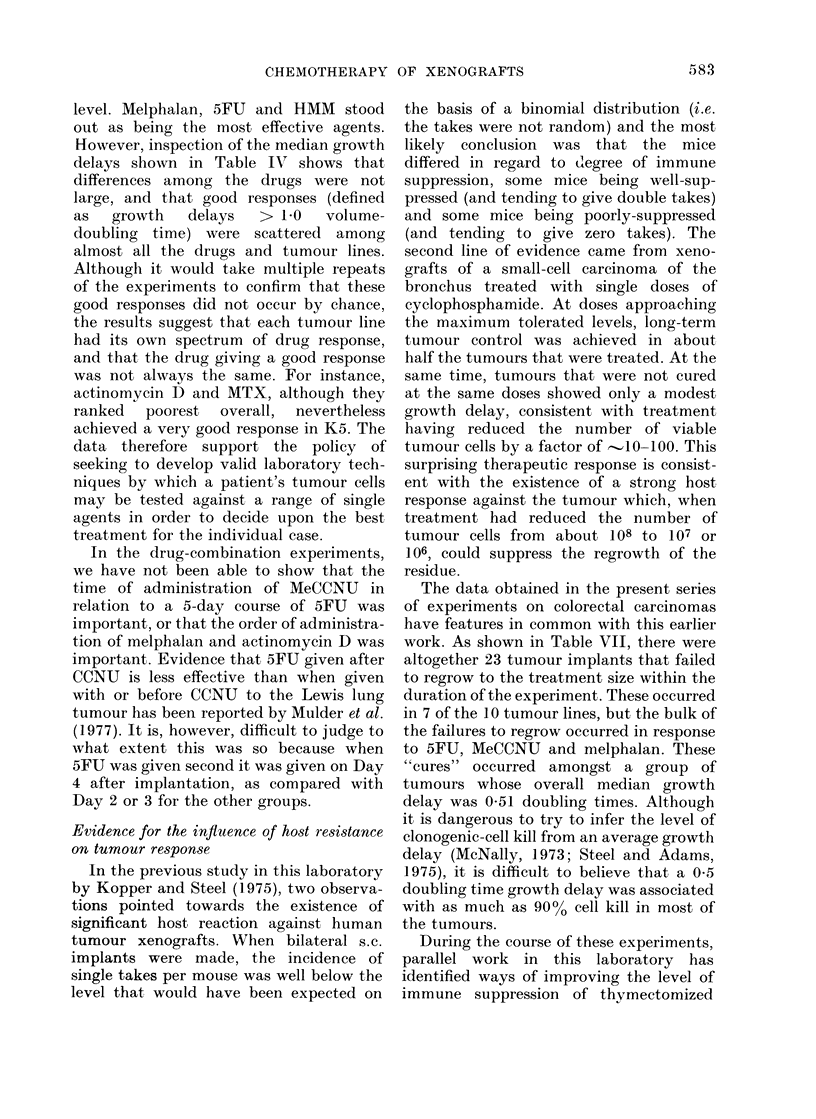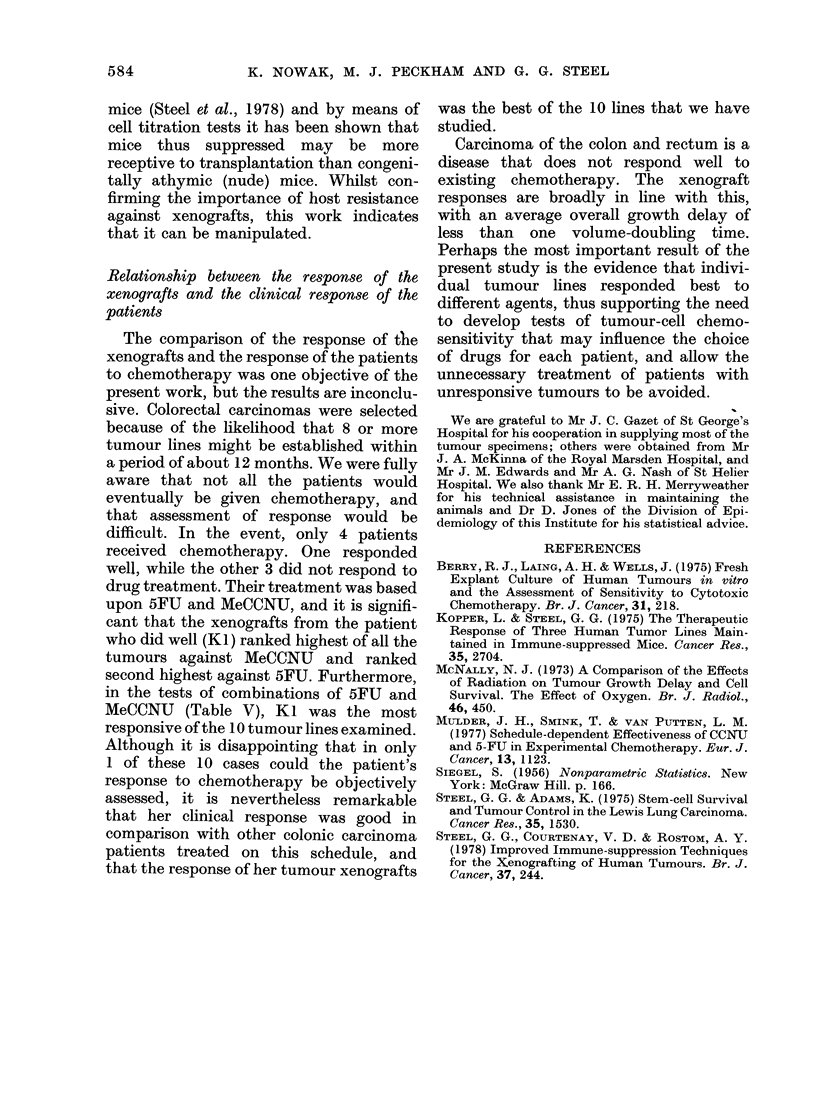# Variation in response of xenografts of colo-rectal carcinoma to chemotherapy.

**DOI:** 10.1038/bjc.1978.87

**Published:** 1978-04

**Authors:** K. Nowak, M. J. Peckham, G. G. Steel

## Abstract

Ten xenograft lines of human colonic and rectal carcinomas have been established in immune-suppressed mice. Mice bearing tumours in the 2nd to 6th passage were treated with maximum tolerated doses of 8 chemotherapeutic agents and tumour growth delay was estimated in terms of the number of volume doubling times gained by the treatment. The average response corresponded to a delay of only about 0.5 doubling times, but some tumour lines showed a good response to some agents. Twenty-three out of about 700 treated tumours failed to regrow. Statistical analysis showed no consistent difference in sensitivity among the tumour lines, but melphalan, 5-fluorouracil and hexamethylmelamine stood out as the most effective agents. A study of two-drug combinations showed that their order of administration had little effect on response. Only 4 of the patients who donated the xenografts were treated with chemotherapy, but among these there was some evidence that response in the xenografts correlated with response in the patient.


*Br. J. Cancer* (1978) **37**, 576

### VARIATION IN RESPONSE OF XENOGRAFTS OF COLO-RECTAL CARCINOMA TO CHEMOTHERAPY

K. NOWAK,* M. J. PECKHAM AND G. G. STEEL†

*From the Radiotherapy Research Unit, Division of Radiotherapy and Biophysics, Institute of Cancer Research, Sutton, Surrey SM2 5PX*

Received 15 November 1977 Accepted 12 December 1977

**Summary.—Ten xenograft lines of human colonic and rectal carcinomas have been established in immune-suppressed mice. Mice bearing tumours in the 2nd to 6th passage were treated with maximum tolerated doses of 8 chemotherapeutic agents and tumour growth delay was estimated in terms of the number of volume doubling times gained by the treatment. The average response corresponded to a delay of only about 0·5 doubling times, but some tumour lines showed a good response to some agents. Twenty-three out of about 700 treated tumours failed to regrow. Statistical analysis showed no consistent difference in sensitivity among the tumour lines, but melphalan, 5-fluorouracil and hexamethylmelamine stood out as the most effective agents. A study of two-drug combinations showed that their order of administration had little effect on response. Only 4 of the patients who donated the xenografts were treated with chemotherapy, but among these there was some evidence that response in the xenografts correlated with response in the patient.**

A QUESTION that is central to the chemotherapy of cancer is to what extent the patients within one particular histopathological disease category differ in regard to the sensitivity of their tumour cells to cytotoxic agents. Attempts have been made to test the chemosensitivity of tumour cells from individual patients, using tissue culture techniques (*e.g.* Berry *et al.*, 1975) but the artificiality of the conditions of exposure and of the biological end-points has meant that the extent of *variation* in response has been particularly difficult to assess. As part of a wider programme of research into the therapeutic response of human tumour xenografts, we have attempted to measure the range of responses among a group of 10 different lines of colo-rectal carcinoma (numbered HXK1–10), and where possible we have sought to correlate the results with the clinical response of the patients from whom the grafts were taken. This paper describes the results of this study, with emphasis on the assessment of inter-patient and inter-drug variations.

### MATERIALS AND METHODS

*Tumour material.*—The tumour tissues used in this study were operative specimens taken from patients with adenocarcinoma of the colon or rectum (Table I). One of the authors (K.N.) personally attended surgery in every case, and examined the tumours *in situ* together with the attending surgeon. Following resection of the tumour, the specimen was washed in sterile saline and a wedge-shaped sample of the growth was taken, including the invading edge. Wherever possible, specimens of liver metastases and involved lymph nodes were also taken. They were transported to the laboratory in ice-cold tissue-culture medium containing antibiotics (Eagle's basal medium (BME) plus penicillin 0·25 g/l, streptomycin 0·05 g/l, neomycin 0·1 g/l). Fragments measuring ~2 mm across were dissected from the most

* Present address: Department of Surgery, St George's Hospital, Tooting, London SW17 0QJ. This work forms part of an M. Phil. thesis submitted by K.N. to the University of London.

† Communications regarding this paper should be directed to Dr Steel.

TABLE I.—*Source of Tumour Material*

| Designation | Patient Sex | Patient Age | Diagnosis | Histological type of adenocarcinoma: Differentiation | Histological type of adenocarcinoma: Other features |
|---|---|---|---|---|---|
| HXK1* | F | 57 | Ca caecum Duke C | Moderate | Mucus-secreting in areas, liver involvement |
| HXK2 | M | 92 | Ca sigmoid Duke C | Moderate | Liver involvement |
| HXK3 | M | 70 | Ca caecum Duke C | Moderate | Liver involvement |
| HXK4 | F | 59 | Ca rectosigmoid Duke C | Moderate | Mucus-secreting |
| HXK5 | F | 73 | Ca caecum Duke C | Poor | |
| HXK6 | M | 69 | Ca rectum Duke B | Moderate | |
| HXK7 | M | 71 | Ca rectum Dike C | Moderate to poor | |
| HXK8 | F | 52 | Ca sigmoid Duke B | Moderate | |
| HXK9 | F | 50 | Ca caecum Duke C | Poor | |
| HXK10 | M | 38 | Ca transverse colon Duke C | Moderate | Mucus-secreting |

* For the sake of clarity, the initial letters HX are omitted from the textual references of these tumours.

viable part of the tumour specimen and implanted s.c. into recipient mice within 2 h of removal from the patient.

*Immune-deprived mice.*—Male and female CBA/lac mice were used, produced in the Institute of Cancer Research breeding station. At 3–4 weeks of age they were thymectomized, and 2 weeks later they were given a dose of 9 gray whole-body irradiation from a $^{60}$Co source, using a dose rate of about 0·65 gray/min. Within 2–4 h the mice received an i.v. injection of $5 \times 10^6$ syngeneic marrow cells to restore haemopoiesis. The mice were kept under non-sterile conditions in a conventional animal house, though separated from rooms containing mice used for non-xenograft work.

*Tumour transplantation and measurement.*—Tumour specimens were placed in chilled BME containing antibiotics, and initially cleared of necrotic tissue and fat. Pieces measuring ~8 mm$^3$ were then removed using sharp scalpels, so as far as possible to preserve uncrushed edges. The pieces used for transplantation were then picked at random and implanted s.c. over the posterior rib cage on both sides of the animal. The implants were inserted with a pair of forceps into deep subcutaneous pockets and each wound closed with a single metal clip. The mice were observed for signs of tumour growth, and shaved to allow the implants to be measured accurately. Further transplantation was carried out from tumours that had reached a volume of ~2 cm$^3$.

Growing tumours were measured every 2–3 days using plastic calipers graduated to 0·1 mm. The largest and smallest superficial dimensions were recorded, and tumour volume was calculated as $\pi/6$ (mean diameter)$^3$. Groups of tumours were selected for chemotherapy when their average volume was ~0·2–0·5 cm$^3$. Volume measurements were continued, and the interval during which each tumour increased to twice its volume at the time of treatment was found by interpolation on a semi-logarithmic plot. For each group of treated or control tumours, the median time to double (TD) was calculated and the growth delay that resulted from each treatment was defined as

$$\text{Growth delay} = \frac{TD_{\text{treated}} - TD_{\text{control}}}{TD_{\text{control}}}$$

This quantity may be regarded as an estimate of the number of volume doubling times saved by the treatment. By calculating in this way, one arrives at a quantity that should allow comparisons to be made between tumours that have different rates of untreated growth.

*Chemotherapy.*—Table II lists the 8 chemotherapeutic agents used in this study. The decision to use 4 agents in single dose and 4 as 5 daily doses was based on the clinical protocols in use in the Royal Marsden Hospital and, in the case of HMM and cis-platinum, on the advice of our colleagues who are using these new agents. The dose levels were selected on the basis of toxicity studies. We attempted to use the $LD_{10}$ level, but because of the notorious variability in maximum-tolerated dose from one experiment to another there was considerable variation in the deaths due to our chosen drug levels.

Two series of combination studies were performed, using 5FU and MeCCNU, and actinomycin D and melphalan. In each case the combination was more toxic than the

TABLE II.—*Chemotherapeutic Agents and Dosage*

| Agent | Manufacturer | Preparation | Dosage* |
|---|---|---|---|
| 5-Fluorouracil (5FU) | Roche Products Ltd. | Aqueous solution | 30 mg/kg qd × 5 |
| MeCCNU | Division of Cancer Treatment, NCI. NSC 95441 | In dimethylsulphoxide and 5% Tween 80 in saline | 30 mg/kg single dose |
| Melphalan (Melph) | Burroughs Wellcome Co. | Ethyl alcohol acidified and propylene glycol in water | 12 mg/kg single dose |
| Actinomycin D (Act D) | Merck, Sharp & Dohme | Aqueous solution | 0·5 mg/kg single dose |
| Hexamethylmelamine (HMM) | Chester Beatty Research Institute | Suspension in arachis oil | 5 mg/kg qd × 5 |
| Methotrexate (MTX) | Lederle Laboratories | Aqueous solution | 5 mg/kg qd × 5 |
| Cyclophosphamide (CY) | W. B. Pharmaceuticals Ltd. | Aqueous solution | 200 mg/kg single dose |
| Cis-dichlorodiamino-platinum (Pt) | Chester Beatty Research Institute | Aqueous solution | 3 mg/kg qd × 5 |

* All given i.p.

drugs used alone. On the basis of toxicity studies, the drug doses were reduced as follows: MeCCNU to 25 mg/kg when given during or after the 5FU course, and to 20 mg/kg when given before the 5FU; the 5FU dose was not reduced. Melphalan was reduced to 8 mg/kg, and actinomycin D to 0·25 mg/kg when given after melphalan and to 0·35 mg/kg when given before melphalan.

FIG.—Example of a set of growth curves for control tumours (top) and treated tumours (bottom). Arrows indicate treatment. This example presents the data for the K4 line treated with 5FU alone.

RESULTS

### *Growth delay in the xenografts*

Within both the treated and untreated groups of tumours there was a range of growth rates and, bearing in mind that this study involved the measurement of over 700 individual treated tumours, it is impossible to present the detailed results on each of them. The Fig. is an example of the data that were obtained. From plots of this type the individual time to double (TD) was measured and the median TD of each treated group was determined. The reason for choosing the median TD was that some tumours failed to regrow during the course of the experiment, and their individual TD values were therefore indefinitely large. The view will be developed below that these failures to regrow probably indicate the combination of a relatively good anti-tumour effect on the part of the agent, with a considerable degree of help from host factors. If this view is correct, we should not give the failures to regrow undue weight, but to exclude these tumours would bias the results *against* tumour lines that showed failures to regrow. In this situation it would seem best to work in terms of median values. Growth delay (as defined above) was calculated using for reference

TABLE III.—*Proportion of Positive Takes*

| Xenograft | Passage | TD* (days) | Animals grafted | No. with tumour(s) | Proportion of mice developing tumour(s) |
|---|---|---|---|---|---|
| K1 | II | 13 | 22 | 16 | |
| | III | 15 | 41 | 35 | 0·82 |
| | IV | 14·5 | 24 | 20 | |
| | V | | 34 | 28 | |
| K2 | II | 18·5 | 60 | 49 | |
| | III | 14 | 13 | 13 | |
| | IV | 14 | 25 | 16 | 0·78 |
| | V | | 24 | 19 | |
| | VI | | 10 | 6 | |
| K3 | II | 19 | 22 | 12 | |
| | III | 16·5 | 30 | 14 | 0·44 |
| | IV | 36 | 25 | 8 | |
| K4 | II | 13 | 50 | 39 | 0·82 |
| | IV | 12·5 | 24 | 22 | |
| K5 | II | 9 | 59 | 53 | 0·89 |
| | III | 10 | 16 | 14 | |
| K6 | II | 13 | 40 | 34 | 0·86 |
| | III | 9·5 | 16 | 14 | |
| K7 | II | 16 | 47 | 47 | 0·97 |
| | III | 14·5 | 20 | 18 | |
| K8 | II | 10·5 | 47 | 33 | 0·53 |
| | III | | 28 | 7 | |
| K9 | II | 11 | 48 | 31 | 0·58 |
| | III | | 18 | 6 | |
| K10 | II | 21·5 | 48 | 40 | 0·83 |

* Median volume-doubling time of untreated controls.

the TD of untreated control tumours implanted at the same time. These TD values are summarized in Table III, together with the proportion of mice grafted with bilateral tumours in which at least one tumour was available for chemotherapeutic investigation. These take-rates per mouse were generally in excess of 50%, but only K7 came close to 100%.

The median growth delays calculated for each group of treated tumours are summarized in Tables IV, V and VI, and Table VII shows the incidence of failures to regrow amongst the various tumour-drug combinations.

*Analysis of the xenograft response to single agents*

Table IV includes growth-delay data for each of the 10 tumour lines challenged with up to 8 single agents. Many of the values are small, but values exceeding 1·0 doubling times indicate a considerable* growth delay, and are given in heavy type. It can be seen that in spite of only 14/64 median growth delays exceeding 1·0, this level of response was achieved at least once with every tumour line, and every drug except cyclophosphamide and cis-platinum achieved this level in at least one tumour line. This simple observation therefore points to the fact that good responses were widely scattered, both among the drugs and among the tumour lines.

The questions that one would hope to answer on the basis of the data given in Table IV are:

(i) Can we say that some tumours showed significantly different response from others?

(ii) What is the ranking of the drugs against these tumours, and are their differences in effectiveness statistically significant?

(iii) Is there evidence for particular tumours showing strong response to particular agents?

The first two of these questions suggest an analysis of variance among the drugs and the tumour lines, and in this situation it is appropriate to use a non-parametric method. We have therefore employed Friedman's two-way analysis of variance by ranks (Siegel, 1956) using median growth delay as the response parameter. The results are shown in Tables VIII and IX. Since some tumour-drug combinations were not studied, this test was applied to 7 drugs and 8 tumour lines.

When the tumour lines are ranked against the drugs (Table VIII) the probability that the sets of ranks occurred by chance on the null hypothesis of no difference among the tumour lines is 0·4. The inter-tumour differences in the ranks are therefore not significant and we must conclude that the tumour lines

* The coefficient of variation of the control TDs was always in the range 0·25–0·35.

TABLE IV.—*Response* of Tumour Xenograft Lines to Single Agents*

| Tumour line | Drug | | | | | | | | Grand median growth delay |
|---|---|---|---|---|---|---|---|---|---|
| | 5FU | MeCCNU | Melph | Act D | HMM | MTX | CY | Pt | |
| K1 | **1·4** | **>5** | **2·5** | 0·5 | 0·9 | 0·2 | 0 | 0·7 | 0·9 |
| K2 | 0·2 | 0·6 | **1·0** | 0 | 0·8 | 0 | 0·1 | (0) | 0·2 |
| K3 | **1·7** | 0·6 | | (0·5) | | | | | |
| K4 | 0·5 | 0 | 0·3 | 0·5 | **1·5** | 0·1 | 0·2 | | 0·4 |
| K5 | **>9** | 0·4 | 0·7 | **2·7** | 0·5 | **2·9** | 0·6 | 0 | 0·7 |
| K6 | 0·6 | 0·4 | 0·8 | 0·1 | **1·4** | 0·7 | 0·5 | | 0·6 |
| K7 | 0·4 | 0·1 | **1·1** | 0·1 | **1·2** | 0·2 | 0·4 | 0·5 | 0·4 |
| K8 | **1·9** | 0·5 | **1·8** | 0·5 | | | | | |
| K9 | 0·5 | 0·1 | (3) | 0·6 | 0·5 | 0·1 | 0·1 | 0·3 | 0·4 |
| K10 | 0·3 | 0·9 | 0·5 | 0·1 | 0·8 | 0·3 | (4·5) | | 0·4 |
| Grand median growth delay | 0·5 | 0·4 | 1·0 | 0·2 | 0·8 | 0·2 | 0·3 | | |

* The figures indicate the median growth delay of each batch of treated tumours. Values in brackets are uncertain, being based on too few tumours. Values in heavy type, growth delays of 1·0 or more.

did not show significant differences in response to all the agents. When, however, the drugs are ranked against the tumours (Table IX) the differences are just significant at the 0·05 level. There is therefore some evidence that the drugs varied in their effectiveness against all the tumour lines. Inspection of the rank totals shows that the scores for melphalan, HMM and 5FU were considerably lower than for the other 4 drugs, and therefore there are grounds for concluding that these 3 agents were significantly more effective than the others. These were the 3 agents that also rank best in terms of the number of tumour lines that gave a growth delay in excess of 1·0 (Table IV).

TABLE V.—*Response* of Tumour Xenograft Lines to Drug Combinations*

| Tumour line | Combination of 5FU and MeCCNU† | | | Combination of Melphalan and Act D‡ | |
|---|---|---|---|---|---|
| | 5FU first | Together | 5FU second | Melph first | Melph second |
| K1 | **>9** | **>7** | **>6** | | **3·0** |
| K2 | 0·7 | 0·5 | 0·4 | (1·8) | 0·3 |
| K3 | **2** | **2·5** | **1·2** | | **1·0** |
| K4 | 0·4 | **1·3** | 0·7 | 0·1 | 0·4 |
| K5 | **2·2** | **>9** | 0·5 | (3) | 0·7 |
| K6 | 0·4 | **1·5** | 0·4 | 0·1 | 0·3 |
| K7 | **1·0** | **1·1** | **1·3** | **1·0** | **1·2** |
| K8 | **1·2** | **1·5** | **1·5** | **1·1** | 0·8 |
| K9 | 0·9 | 0·7 | **1·8** | **1·1** | 0·5 |
| K10 | 0·5 | 0·4 | (1) | 0·6 | **1·8** |
| Grand median growth delay | 1·0 | 1·4 | 1·2 | 1·0 | 0·8 |

* The figures indicate the median growth delay of each batch of treated tumours. Values in heavy type, growth delays of 1·0 or more.

† 5FU given in 5 daily doses. MeCCNU given 5 days before the first, with the third, or 5 days after the fifth dose. MeCCNU dose was reduced from 25 mg/kg to 20 mg/kg when given before 5FU.

‡ Melphalan was followed by Act D (0·25 mg/kg) at 1 day; Act D (0·35 mg/kg) was followed by Melphalan at 1 h.

TABLE VI.—*Response* of Tumour Xenograft Lines to Combinations of Melphalan and Act D*

| Tumour line | Combination | |
|---|---|---|
| | Melph→Act D† | Act D→Melph‡ |
| K1 | | **3·0** |
| K2 | (1·8) | 0·3 |
| K3 | | **1·0** |
| K4 | 0·1 | 0·4 |
| K5 | (3) | 0·7 |
| K6 | 0·1 | 0·3 |
| K7 | **1·0** | **1·2** |
| K8 | **1·1** | 0·8 |
| K9 | **1·1** | 0·5 |
| K10 | 0·6 | **1·8** |
| Grand median growth delay | 1·0 | 0·8 |

* The figures indicate the median growth delay of each batch of treated tumours. Values in heavy type, growth delays of 1·0 or more.

† Melphalan was followed by Act D (0·25 mg/kg) at 1 day.

‡ Act D (0·35 mg/kg) was followed by melphalan at 1 h.

TABLE VII.—*Incidence of Tumours That Failed to Regrow**

| Tumour line | Drug: 5FU | MeCCNU | Melph | Act D | HMM | MTX | CY | Pt | Total failures to regrow |
|---|---|---|---|---|---|---|---|---|---|
| K1 | | 5/9 | 1/4 | | | | | | 6 |
| K2 | 2/8 | | | | | | | | 2 |
| K3 | 1/9 | | | | | | | | 1 |
| K4 | | | 2/6 | | 1/6 | | | | 3 |
| K5 | 4/6 | 2/8 | | | | 1/5 | | | 7 |
| K6 | | | | | | | | | 0 |
| K7 | | | 2/6 | | | | | | 2 |
| K8 | | | | | | | | | 0 |
| K9 | | | 2/4 | | | | | | 2 |
| K10 | | | | | | | | | 0 |
| Total failures to regrow | 7 | 7 | 7 | 0 | 1 | 1 | 0 | 0 | 23 |

* Tumours that failed to return to treatment size within the duration of the experiment (2 months or more) as a fraction of the number of tumours whose size was followed.

TABLE VIII.—*Statistical Analysis* of Tumour Ranking*

| | 5FU | MeCCNU | Melph | Act D | HMM | MTX | CY | Rank total |
|---|---|---|---|---|---|---|---|---|
| K1 | 2 | 1 | 1 | 4 | 4 | 4·5 | 8 | 24·5 |
| K2 | 8 | 3 | 4 | 8 | 5·5 | 8 | 6·5 | 43 |
| K3 | | | | | | | | |
| K4 | 4·5 | 8 | 8 | 3 | 1 | 6·5 | 5 | 36 |
| K5 | 1 | 4·5 | 6 | 1 | 7·5 | 1 | 2 | 23 |
| K6 | 3 | 4·5 | 5 | 7 | 2 | 2 | 3 | 26·5 |
| K7 | 6 | 6·5 | 3 | 6 | 3 | 4·5 | 4 | 33 |
| K8 | | | | | | | | |
| K9 | 4·5 | 6·5 | 2 | 2 | 7·5 | 6·5 | 6·5 | 35·5 |
| K10 | 7 | 2 | 7 | 5 | 5·5 | 3 | 1 | 30·5 |

$\chi^2 = 7{\cdot}6$ (7 d.f.). $P = 0{\cdot}4$.

* By the Friedman 2-way analysis of variance by ranks (Siegel, 1956). Low ranks indicate *good* response.

TABLE IX.—*Statistical Analysis* of Drug Ranking*

| | 5FU | MeCCNU | Melph | Act D | HMM | MTX | CY |
|---|---|---|---|---|---|---|---|
| K1 | 3 | 1 | 2 | 5 | 4 | 6 | 7 |
| K2 | 4 | 3 | 1 | 6·5 | 2 | 6·5 | 5 |
| K3 | | | | | | | |
| K4 | 2·5 | 7 | 4 | 2·5 | 1 | 6 | 5 |
| K5 | 1 | 7 | 4 | 3 | 6 | 2 | 5 |
| K6 | 4 | 6 | 2 | 7 | 1 | 3 | 5 |
| K7 | 3·5 | 6·5 | 2 | 6·5 | 1 | 5 | 3·5 |
| K8 | | | | | | | |
| K9 | 3·5 | 6 | 1 | 2 | 3·5 | 6 | 6 |
| K10 | 5·5 | 2 | 4 | 7 | 3 | 5·5 | 1 |
| Rank total | 27 | 38·5 | 20 | 39·5 | 21·5 | 40 | 37·5 |

$\chi^2 = 12{\cdot}6$ (6 d.f.).
$P = 0{\cdot}05$.

* By the Friedman 2-way analysis of variance by ranks (Siegel, 1956). Low ranks indicate *good* response.

Although there does appear to be evidence for significant differences in drug effectiveness, it should not be overlooked that the less effective agents did give good responses with *some* tumour lines (Table IV). The data therefore support the view that, over and above the broad ranking of drugs, there was evidence for the specific sensitivity of a given tumour to one or more particular drugs.

*Analysis of the xenograft response to drug combinations*

The studies of drug combinations were broadly disappointing. The choice of 2 pairs of drugs (5FU and MeCCNU; melphalan and actinomycin D) was made at the start of the investigation, before the single-agent ranking was known. In the event, each of these combinations included one high-ranking drug and one that ranked poorly. The objective of the combination studies was not to show that the combinations could give a greater tumour growth delay than single agents (to show this would require proof that the combination and single-agent treatments were equitoxic). The objective was to study the effect of timing in the drug combinations. It is from this point of view that the results are disappointing: the study has probably failed to identify an optimum drug timing. As shown in Tables V and VI, each combination gave a good proportion of responses in excess of 1·0 doubling times. In K1 and K5 treated with 5FU and melphalan, there were treatment groups in which more than half the tumours failed to regrow during the course of the experiment. In these groups it is not possible to obtain the median growth delay, so a minimum value is given instead. It can be seen that these median values show no obvious trend in favour of one combination over another and the grand median values for each combination against all the tumour lines are similar.

*Clinical response of the patients*

Of the 10 patients from whom xenograft lines were established, 4 were treated with cytotoxic drugs but subsequently died. The remaining 6 received no treatment other than surgery and 2 of these patients are alive and well. The patients who received chemotherapy were the donors of xenografts K1, K3, K9 and K10.

*K1 patient*

During an operation for right hemicolectomy the liver of this patient was found to contain multiple metastases. On abdominal examination, the edge of the liver could be palpated below the right costal margin, and ultrasonography confirmed the presence of multiple lesions. The serum carcinoembryonic antigen (CEA) level was elevated (62 ng/ml). The course of treatment consisted of MeCCNU (150 mg/m$^2$ on Day 1), 5FU (325 mg/m$^2$ i.v. daily for 5 consecutive doses), and imidazole carboxamide (75 mg/m$^2$ i.v. daily for 5 consecutive doses). Eight courses of treatment were given over a period of 11 months.

About 2 months after the start of treatment, the condition of the patient improved, she began to gain weight and the serum CEA level fell. The liver was no longer palpable and the ultrasound scan showed no progression of the disease. In spite of continued treatment, 5 months later the patient's condition deteriorated and she died, having survived 14 months from diagnosis.

*K3 patient*

This patient had a palliative right hemicolectomy. The liver was enlarged and multiple liver metastases were found; the serum CEA level was 480 ng/ml. This patient received 4 courses of chemotherapy (as for patient K1) but this was discontinued when it became evident that he was not responding. The survival time from diagnosis was 13 months.

*K9 and K10 patients*

Both these patients had rapidly progressing disease. There was no evidence of response to chemotherapy and they died having received only one and 3 courses of treatment respectively.

### DISCUSSION

*General conclusions on the ranking of the anti-tumour agents*

The statistical test that is summarized in Table IX has shown that, when the drugs are ranked in terms of the response achieved with all the 8 tumour lines that could be assessed, the differences in response were just significant at the 0·05

level. Melphalan, 5FU and HMM stood out as being the most effective agents. However, inspection of the median growth delays shown in Table IV shows that differences among the drugs were not large, and that good responses (defined as growth delays $> 1{\cdot}0$ volume-doubling time) were scattered among almost all the drugs and tumour lines. Although it would take multiple repeats of the experiments to confirm that these good responses did not occur by chance, the results suggest that each tumour line had its own spectrum of drug response, and that the drug giving a good response was not always the same. For instance, actinomycin D and MTX, although they ranked poorest overall, nevertheless achieved a very good response in K5. The data therefore support the policy of seeking to develop valid laboratory techniques by which a patient's tumour cells may be tested against a range of single agents in order to decide upon the best treatment for the individual case.

In the drug-combination experiments, we have not been able to show that the time of administration of MeCCNU in relation to a 5-day course of 5FU was important, or that the order of administration of melphalan and actinomycin D was important. Evidence that 5FU given after CCNU is less effective than when given with or before CCNU to the Lewis lung tumour has been reported by Mulder *et al.* (1977). It is, however, difficult to judge to what extent this was so because when 5FU was given second it was given on Day 4 after implantation, as compared with Day 2 or 3 for the other groups.

*Evidence for the influence of host resistance on tumour response*

In the previous study in this laboratory by Kopper and Steel (1975), two observations pointed towards the existence of significant host reaction against human tumour xenografts. When bilateral s.c. implants were made, the incidence of single takes per mouse was well below the level that would have been expected on the basis of a binomial distribution (*i.e.* the takes were not random) and the most likely conclusion was that the mice differed in regard to degree of immune suppression, some mice being well-suppressed (and tending to give double takes) and some mice being poorly-suppressed (and tending to give zero takes). The second line of evidence came from xenografts of a small-cell carcinoma of the bronchus treated with single doses of cyclophosphamide. At doses approaching the maximum tolerated levels, long-term tumour control was achieved in about half the tumours that were treated. At the same time, tumours that were not cured at the same doses showed only a modest growth delay, consistent with treatment having reduced the number of viable tumour cells by a factor of ~10–100. This surprising therapeutic response is consistent with the existence of a strong host response against the tumour which, when treatment had reduced the number of tumour cells from about $10^8$ to $10^7$ or $10^6$, could suppress the regrowth of the residue.

The data obtained in the present series of experiments on colorectal carcinomas have features in common with this earlier work. As shown in Table VII, there were altogether 23 tumour implants that failed to regrow to the treatment size within the duration of the experiment. These occurred in 7 of the 10 tumour lines, but the bulk of the failures to regrow occurred in response to 5FU, MeCCNU and melphalan. These "cures" occurred amongst a group of tumours whose overall median growth delay was 0·51 doubling times. Although it is dangerous to try to infer the level of clonogenic-cell kill from an average growth delay (McNally, 1973; Steel and Adams, 1975), it is difficult to believe that a 0·5 doubling time growth delay was associated with as much as 90% cell kill in most of the tumours.

During the course of these experiments, parallel work in this laboratory has identified ways of improving the level of immune suppression of thymectomized mice (Steel *et al.*, 1978) and by means of cell titration tests it has been shown that mice thus suppressed may be more receptive to transplantation than congenitally athymic (nude) mice. Whilst confirming the importance of host resistance against xenografts, this work indicates that it can be manipulated.

*Relationship between the response of the xenografts and the clinical response of the patients*

The comparison of the response of the xenografts and the response of the patients to chemotherapy was one objective of the present work, but the results are inconclusive. Colorectal carcinomas were selected because of the likelihood that 8 or more tumour lines might be established within a period of about 12 months. We were fully aware that not all the patients would eventually be given chemotherapy, and that assessment of response would be difficult. In the event, only 4 patients received chemotherapy. One responded well, while the other 3 did not respond to drug treatment. Their treatment was based upon 5FU and MeCCNU, and it is significant that the xenografts from the patient who did well (K1) ranked highest of all the tumours against MeCCNU and ranked second highest against 5FU. Furthermore, in the tests of combinations of 5FU and MeCCNU (Table V), K1 was the most responsive of the 10 tumour lines examined. Although it is disappointing that in only 1 of these 10 cases could the patient's response to chemotherapy be objectively assessed, it is nevertheless remarkable that her clinical response was good in comparison with other colonic carcinoma patients treated on this schedule, and that the response of her tumour xenografts was the best of the 10 lines that we have studied.

Carcinoma of the colon and rectum is a disease that does not respond well to existing chemotherapy. The xenograft responses are broadly in line with this, with an average overall growth delay of less than one volume-doubling time. Perhaps the most important result of the present study is the evidence that individual tumour lines responded best to different agents, thus supporting the need to develop tests of tumour-cell chemosensitivity that may influence the choice of drugs for each patient, and allow the unnecessary treatment of patients with unresponsive tumours to be avoided.

We are grateful to Mr J. C. Gazet of St George's Hospital for his cooperation in supplying most of the tumour specimens; others were obtained from Mr J. A. McKinna of the Royal Marsden Hospital, and Mr J. M. Edwards and Mr A. G. Nash of St Helier Hospital. We also thank Mr E. R. H. Merryweather for his technical assistance in maintaining the animals and Dr D. Jones of the Division of Epidemiology of this Institute for his statistical advice.

### REFERENCES

BERRY, R. J., LAING, A. H. & WELLS, J. (1975) Fresh Explant Culture of Human Tumours *in vitro* and the Assessment of Sensitivity to Cytotoxic Chemotherapy. *Br. J. Cancer*, **31**, 218.

KOPPER, L. & STEEL, G. G. (1975) The Therapeutic Response of Three Human Tumor Lines Maintained in Immune-suppressed Mice. *Cancer Res.*, **35**, 2704.

McNALLY, N. J. (1973) A Comparison of the Effects of Radiation on Tumour Growth Delay and Cell Survival. The Effect of Oxygen. *Br. J. Radiol.*, **46**, 450.

MULDER, J. H., SMINK, T. & VAN PUTTEN, L. M. (1977) Schedule-dependent Effectiveness of CCNU and 5-FU in Experimental Chemotherapy. *Eur. J. Cancer*, **13**, 1123.

SIEGEL, S. (1956) *Nonparametric Statistics.* New York: McGraw Hill. p. 166.

STEEL, G. G. & ADAMS, K. (1975) Stem-cell Survival and Tumour Control in the Lewis Lung Carcinoma. *Cancer Res.*, **35**, 1530.

STEEL, G. G., COURTENAY, V. D. & ROSTOM, A. Y. (1978) Improved Immune-suppression Techniques for the Xenografting of Human Tumours. *Br. J. Cancer*, **37**, 244.